# 244 Integrating WGS with Epidemiologic Data to Guide Containment in a Multi-Facility CP-CRAB Outbreak — Georgia, Nov 2024–May 2025

**DOI:** 10.1017/ash.2026.10616

**Published:** 2026-06-23

**Authors:** Koko Shibutani, Chie Masuda, Yumiko Mikami, Sayaka Fujisaki, Yasuhiro Ozawa, Kazunori Hattori, Nobuyoshi Mori

**Affiliations:** 1 Division of Infectious Diseases, St. Luke’s International Hospital, Tokyo, Japan; 2 St. Luke’s International Hospital; 3 Dentistry and Oral Surgery; 4 Dentistry & Oral Surgery

## Abstract

**Background:** Mycobacteria are widely distributed in the environment, and colonization of medical devices using water poses a risk in healthcare settings. Hospital outbreaks linked to ventilators and extracorporeal systems have been reported. At our hospital, dental unit water is routinely cultured every three months. In January 2024, unexpected mycobacterial detection prompted an investigation of dental unit waterlines, tanks, and water sources. Objective: To investigate the presence of mycobacteria in dental unit waterlines and evaluate clinical implications. **Methods:** Water samples (300ml) from six dental unit waterlines were tested by culture before and after changes in flushing frequency, heater use, and filter replacement. Additional samples from the hospital annex water tanks, main building tank, and nearby parks sharing the same water source were analyzed. **Results:** Mycobacteria were isolated from all six dental unit waterlines, including M. frederiksbergense, M. chelonae complex, M. mageritense, and M. mucogenicum/phocaicum, with concentrations up to 25 CFU/mL. Increasing flushing from weekly to daily and discontinuing heater use did not eliminate contamination. However, quantitative cultures after filter replacement showed marked reduction, with most lines becoming negative or <0.5 CFU/mL. Mycobacteria were also detected in external water sources: annex water tank (10 CFU/mL), main building tank (<2.5 CFU/mL), and nearby parks (<5 CFU/mL). These findings suggest mycobacteria are not unique to dental units but are ubiquitously present in tap water. Discussion: Detection in both dental units and external water sources indicates widespread colonization rather than isolated contamination. Filtration proved effective in significantly reducing mycobacterial load, emphasizing its role in infection control. Given the pathogenic potential of environmental mycobacteria, especially in immunocompromised patients such as those with neutropenia or hematopoietic cell transplantation, exposure via dental unit water may carry clinical risks. For these patients, use of sterile water instead of tap water during dental treatment is strongly considered. **Conclusion:** Mycobacteria are present in tap water and dental unit waterlines. Filter replacement effectively reduces contamination. Strict precautions, including sterile water use, are warranted to protect vulnerable patients from opportunistic mycobacterial infections.

